# Pirtobrutinib in Richter syndrome: Outcomes from the named patient programme in Italy

**DOI:** 10.1111/bjh.70805

**Published:** 2026-08-30

**Authors:** Isacco Ferrarini, Daniela Estefania Banegas, Andrea Bernardelli, Alessia Moioli, Francesca Maria Quaglia, Ester Lovato, Anna Maria Frustaci, Massimiliano Postorino, Davide Facchinelli, Monica Tani, Francesca Cibien, Chandrakala Toldo, Elisa Lucchini, Roberto Marasca, Marzia Varettoni, Chiara Rusconi, Lucia Farina, Andrea Visentin, Riccardo Moia, Carlo Visco

**Affiliations:** ^1^ Hematology Unit, Section of Biomedicine of Innovation, Department of Engineering for Innovative Medicine University of Verona Verona Italy; ^2^ UOC di Ematologia, DAI Medico Generale, AOUI Verona Verona Italy; ^3^ Hematology—ASST Spedali Civili Brescia Italy; ^4^ Department of Haematology Niguarda Cancer Center, ASST Grande Ospedale Metropolitano Niguarda Milan Italy; ^5^ Ematologia, Dipartimento di Biomedicina e Prevenzione Università Degli Studi di Roma “Tor Vergata” Rome Italy; ^6^ Department of Hematology San Bortolo Hospital Vicenza Italy; ^7^ Hematology Ospedale “Santa Maria delle Croci” di Ravenna Ravenna Italy; ^8^ Hematology Unit Ca' Foncello Hospital Treviso Italy; ^9^ U.O. Medicina Interna, Section of Hematology and Stem Cell Transplantation ASUIT Trento Trento Autonomous Province of Trento Italy; ^10^ Hematology Unit Azienda Sanitaria Universitaria Giuliano Isontina Trieste Italy; ^11^ Section of Hematology, Department of Medical and Surgical Sciences University of Modena and Reggio Emilia Modena Italy; ^12^ SC Ematologia 1, Fondazione IRCCS Policlinico San Matteo Pavia Italy; ^13^ Hematology Fondazione IRCCS Istituto Nazionale dei Tumori Milan Italy; ^14^ University of Padua Padova Italy; ^15^ Division of Hematology, Department of Translational Medicine Università del Piemonte Orientale and AOU Maggiore della Carità di Novara Novara Italy

**Keywords:** BTK, pirtobrutinib, Richter syndrome

To the Editor,

Richter syndrome (RS), the clonal evolution of chronic lymphocytic leukaemia (CLL) into a diffuse large B‐cell lymphoma, remains a clinically challenging event within the CLL natural history,^1^ with median overall survival (OS) rarely exceeding 12 months.^2^ Anthracycline‐based chemoimmunotherapy yields overall response rates (ORRs) of 40%–60%. However, median progression‐free survival (PFS) is typically less than 6 months, with substantial haematological toxicity.^3^

Although covalent Bruton tyrosine kinase (cBTK) inhibitors and B‐cell lymphoma 2 (Bcl‐2) antagonists have revolutionized CLL management,^4^ they provide transient disease control in RS. A Mayo Clinic series of four RS patients treated with ibrutinib recorded a 75% ORR, yet median exposure was just 6.1 months and two responders relapsed within a year.^5^ Weak activity was documented also for venetoclax as single agent: in a phase I clinical trial, seven patients with RS had an ORR of 43%, with a median PFS of 1 month.^6^ Moreover, the majority of contemporary RS patients have received either cBTK inhibitors and/or venetoclax for the treatment of the previous CLL phase, further complicating the clinical scenario and highlighting the need for novel therapeutic strategies.

Pirtobrutinib, a non‐cBTK inhibitor with subnanomolar potency against both wild‐type and C481‐mutant BTK, represents a novel strategy to circumvent acquired resistance and to effectively block BTK signalling.^7^ Within the phase 1/2 BRUIN trial, patients with RS had an ORR of 50%, thereby establishing a new therapeutic opportunity for relapsed/refractory (R/R) RS patients.^8^ However, real‐world evidence about the efficacy and safety of pirtobrutinib in RS remains scarce.

To address this gap, we present herein a retrospective analysis of patients with RS treated with pirtobrutinib within the named patient programme in Italy (project approved by local ethics committee, number 523CET). Twenty‐four RS patients (intention‐to‐treat population) were identified across 12 tertiary centres. Two patients received at least one dose of pirtobrutinib but died at day 12 and 15 (one due to disease progression and one of grade 5 infection, respectively) and were not considered for further analyses. The study population encompassed adult patients with RS who received at least one cycle (28 days) of pirtobrutinib between September 2022 and March 2026. The median age at pirtobrutinib initiation was 69 years (range 45–90), and 15 (68%) were male. Eastern Cooperative Oncology Group performance status (ECOG‐PS) 2–3 occurred in 6 (27%) cases. Elevated lactate dehydrogenase (LDH) and bulky nodal masses (≥5 cm) were described in 17 (77%) and 12 (55%) patients respectively. *TP53* status was evaluable in 15 patients (68%), and 9 (60%) harboured *TP53* mutations as assessed by next‐generation sequencing. Patients had received a median of 3.5 (range 1–6) systemic lines across the CLL and RS phases (Table 1). The median number of CLL‐directed and RS‐directed treatments were 2 (range 0–5) and 1 (range 0–3) respectively. Nineteen patients (86%) received chemo‐immunotherapy as first line for the RS phase. One patient with histological transformation occurring after three CLL‐directed therapies and an allogeneic haematopoietic stem cell transplant (HSCT) received pirtobrutinib as the sole treatment for RS. Two additional patients treated with three and four prior lines for CLL, respectively, were deemed unfit for chemotherapy and received pirtobrutinib as first‐line therapy for RS. Fifteen patients (68%) had previously been exposed to a cBTK inhibitor: 12 (55%) during the CLL phase and 3 (14%) during the RS phase. Twelve patients (55%) had been exposed to venetoclax and 11 (50%) were double‐exposed to cBTK inhibitors and venetoclax. Eleven of 15 patients (73%) discontinued cBTK inhibitor because of disease progression, whereas 4 (27%) discontinued due to toxicity. At pirtobrutinib start, 7 patients (32%) were on steroids and 7 (32%) were on antiplatelet or direct oral anti‐coagulant agents. All participants initiated pirtobrutinib at the recommended dose of 200 mg once daily. Response was evaluated as per local physician's clinical decision, by positron emission tomography/computed tomography (PET/CT) or CT scan (following Lugano criteria) in 14 patients (64%), by ultrasound in 3 patients (14%) and by clinical and laboratory examinations in 6 patients (27%). First response assessment was at 1 month after pirtobrutinib start. Responding patients were categorized as short‐lived (response lasting 4 months or less) and long‐term responders (response lasting more than 4 months).

**TABLE 1:** Baseline patient characteristics.

	PIRTO‐Italy (*n* = 22)
Age (range)	69 (45–90)

	*N*	%
Gender		
Male	15	68
Female	7	32
ECOG PS		
0	4	18
1	11	50
2 or 3	6	27
Unknown	1	5
Increased LDH		
Yes	17	77
No	5	23
*TP53* mutation		
Yes	9	41
No	6	27
Unknown	7	32
PET SUV max median	19.8	
Prior therapy for CLL		
Yes	17	77
No	5	23
Type of CLL therapy		
cBTK inhibitor	12	55
Chemotherapy	14	64
BCL‐2 inhibitor	12	55
PI3K inhibitor	2	9
Allogeneic haematopoietic stem cell transplant	1	5
Prior therapy for RS		
Yes	19	86
No	3	14
Type of RS therapy		
cBTK inhibitor	3	14
Chemotherapy	19	86
BCL‐2 inhibitor	0	0
Prior lines of CLL therapy, median (range)	2 (0–5)	
Prior lines of RS therapy, median (range)	1 (0–3)	
Prior lines of CLL and RS therapy, median (range)	3.5 (1–6)	

Abbreviations: cBTK, covalent Bruton tyrosine kinase; CLL, chronic lymphocytic leukaemia; ECOG PS, Eastern cooperative Oncology Group performance status; PI3K, phosphoinositide 3‐kinase; RS, Richter syndrome; SUV, standardized uptake value.

With data cut‐off on March 2026, the median follow‐up since start of pirtobrutinib was 15.8 months (95% CI 14.5 to NR). At the time of the present analysis, 13 patients (59%) had discontinued therapy: 11 due to radiologically confirmed disease progression, one due to consolidative allogeneic HSCT and one due to a grade 5 septic event. The median time on pirtobrutinib was 4.6 months (range 1.0–33.7).

The ORR was 72.7% (16/22), with 13.6% complete response (CR), 45.5% partial response (PR, radiologically confirmed) and 13.6% clinical/laboratory improvement without radiological assessment. Non‐responders represented 27.3% of the study population, with stable disease and primary progressive disease both occurring in 18% of patients (Figure 1A). Kaplan–Meier estimates yielded a median PFS of 6.1 months (95% CI 3.53 to not reached [NR]) and a median OS of 18.2 months (95% CI 4.1 to NR) (Figure 1B,C). Six‐month actuarial PFS and OS were 45% and 52% respectively. In the intention‐to‐treat population, median PFS and OS were 5.7 and 6.0 months respectively (Figure S1A,B). One patient with partial response to pirtobrutinib was able to proceed to allogeneic HSCT with curative intent, and one with progressive disease was temporarily rescued by glofitamab. Notably, approximately one‐third of responding patients (5/16, 31.2%) experienced a short‐lived response, lasting 4 months or less (Figure 1A). The clinical course of a prototypic short‐lived responder is illustrated in Figure S2A. Short‐lived responders were enriched for cBTK‐resistant cases (100% vs. 45% among long‐term responders, *p* = 0.1186) and, importantly, had a median OS comparable to that of non‐responders and significantly worse than that of long‐term responders (short‐lived responders: 3.2 months; non‐responders: 4.1 months; long‐term responders: unreached. *p* < 0.0001) (Figure S2B,C). Seven long‐term responders (31.8%) remained progression free beyond 6 months, with the longest ongoing response censored at 33.7 months (Figure 1A). The three patients with the most durable responses had not received any prior cBTK inhibitor, suggesting the absence of acquired resistance to this drug class may impact favourably on the clinical activity of pirtobrutinib; however, given the small sample size, this finding should be considered exploratory. In the subgroup of patients with evaluable *TP53* status (*N* = 15), no difference in terms of PFS and OS was noted between wild‐type and mutant cases (*p* = 0.42 and *p* = 0.15 respectively).

**FIGURE 1:**
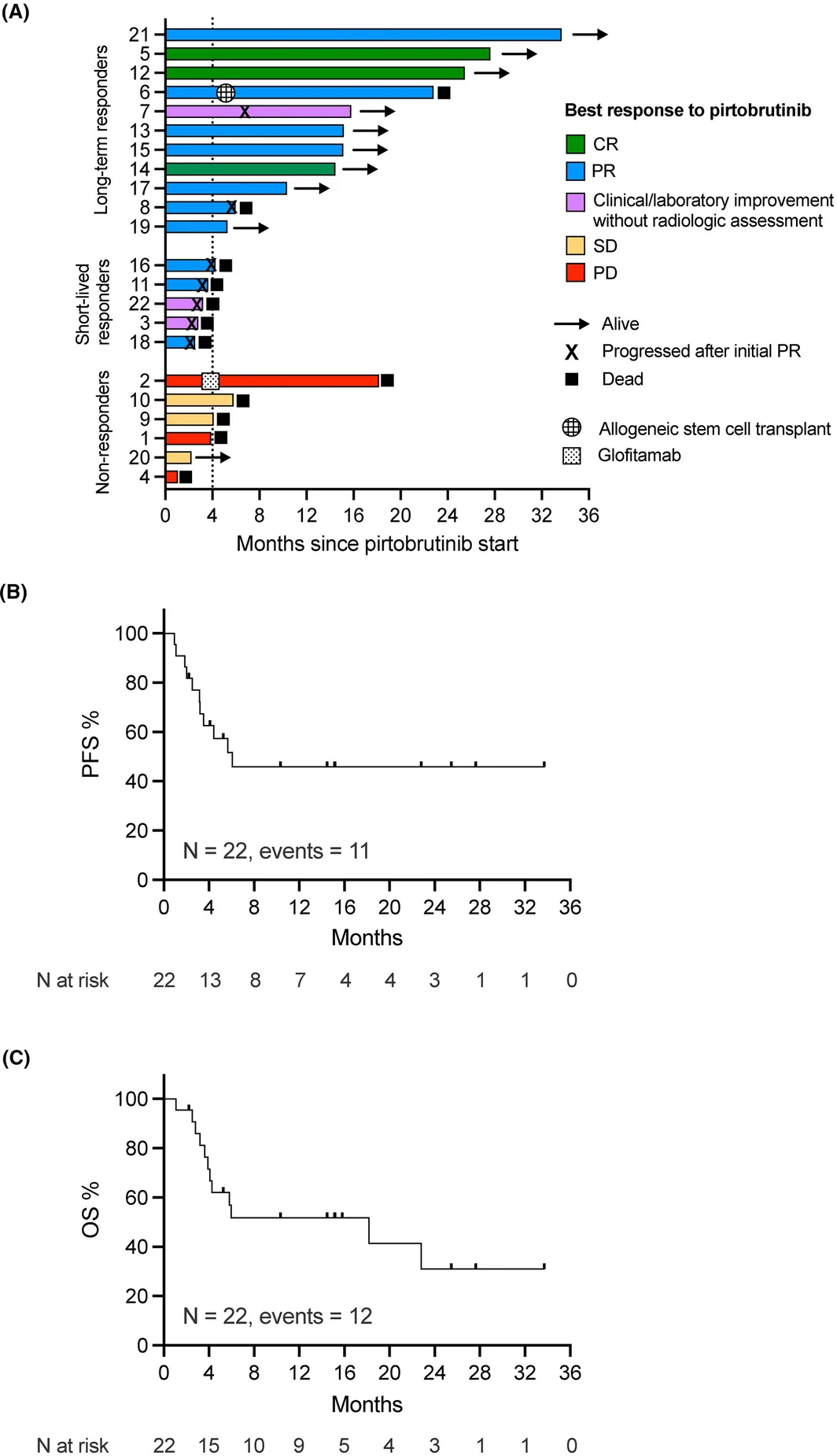
Outcome of RS patients treated with pirtobrutinib. (A) Swimmer's plot of the study patients (*N* = 22), distinguished into long‐term responders (>4 months), short‐lived responders (4 months or less) and non‐responders. Patients alive at last follow‐up are represented by arrowheads; patients who died are represented by black boxes at the end of the bar. Relapse has been denoted by black crosses only for patients who responded to treatment. Time ‘0’ is the first day of pirtobrutinib. CR, complete remission; PD, progression of disease; PR, partial remission; RS, Richter syndrome; SD, stable disease. Patients #7, 22, 3, 1, 20 and 4 did not undergo radiological assessment after pirtobrutinib treatment. In these patients, response was evaluated only by clinical examination and laboratory parameters. (B) Progression‐free survival (PFS). (C) Overall survival (OS).

Tolerability was favourable. A single patient (5%) required permanent dose reduction to 50 mg for grade 3 neutropenia. Two patients temporarily withheld pirtobrutinib: one case due to heart failure deemed unrelated to the drug and another following a concomitant diagnosis of oesophageal neoplasia. No clinically meaningful arrhythmias or major haemorrhagic events were observed. Grade ≥3 haematological adverse events were confined to cytopenias in four patients (18%, 14% neutropenia and 4% thrombocytopenia). Infectious complications occurred in four subjects (18%): three resolved under standard antimicrobial therapy, whereas one sepsis was fatal 4.1 months after pirtobrutinib initiation despite normal neutrophil counts. Such fatal infectious complication occurred in a patient who received prior allogeneic HSCT.

Overall, the outcomes of RS patients in our real‐life cohort confirm substantial single‐agent activity of pirtobrutinib. The ORR observed in our series was higher than that reported in the BRUIN study (72.7% and 50.0% respectively). Likewise, PFS and OS appeared more prolonged in our cohort than in BRUIN (Table S1).^8^ These differences may reflect the lower median number of prior RS‐directed therapies in our cohort compared with BRUIN (1 vs. 2, respectively), which may have reduced the likelihood of selecting shared resistance mechanisms. Moreover, the 2‐month first‐response assessment in BRUIN may have missed very short‐lived clinical responses that were captured in our real‐world study. The lack of radiologic evaluation in six patients, including two categorized as short‐lived partial responders and one categorized as long‐term partial responder based on clinical and laboratory improvement, may also have resulted in an overestimation of response quality. Nevertheless, in these patients and more broadly among short‐lived responders, pirtobrutinib achieved rapid, though transient, disease debulking, supporting its potential role as a backbone for combination approaches. The performance status of our patients was poorer, with an ECOG‐PS ≥2 in 27% and 15% of patients enrolled in our study and BRUIN, respectively, underscoring the feasibility of pirtobrutinib in a frailer patient population.^8^

Additional treatment modalities are being tested in R/R RS, such as the combinations of cBTK inhibitors with chemotherapy and immunotherapy approaches (Table S1).^9^, ^10^, ^11^, ^12^, ^13^, ^14^, ^15^ While cross‐trial comparisons have inherent limitations, the clinical activity of pirtobrutinib seems comparable to that of more toxic and logistically less practical combination strategies. Therefore, pirtobrutinib may help reduce tumour burden in fit patients before they undergo cellular therapies, or it may serve as a definitive treatment for patients who are unfit for more intensive approaches, especially in cases of BTK inhibitor‐naïve disease.

Limitations include the retrospective design, limited follow‐up, absent systematic clonality assessment and small sample size rendering analyses of cBTK inhibitor resistance and *TP53* status underpowered; non‐significant findings therefore cannot exclude outcome associations. Nonetheless, this first real‐world pirtobrutinib series in RS provides a reference for studies of non‐cBTK inhibitors in first‐line and relapsed disease.

## AUTHOR CONTRIBUTIONS

**Davide Facchinelli:** Data curation. **Monica Tani:** Data curation. **Andrea Bernardelli:** Data curation; formal analysis. **Francesca Maria Quaglia:** Data curation. **Anna Maria Frustaci:** Data curation. **Daniela Estefania Banegas:** Data curation; writing – original draft; formal analysis; methodology. **Isacco Ferrarini:** Conceptualization; writing – original draft; supervision; formal analysis; methodology. **Chandrakala Toldo:** Data curation. **Massimiliano Postorino:** Data curation. **Elisa Lucchini:** Data curation. **Marzia Varettoni:** Data curation. **Andrea Visentin:** Data curation. **Francesca Cibien:** Data curation. **Lucia Farina:** Data curation. **Roberto Marasca:** Data curation. **Carlo Visco:** Supervision; writing – review and editing; methodology. **Ester Lovato:** Data curation. **Alessia Moioli:** Data curation. **Chiara Rusconi:** Data curation. **Riccardo Moia:** Data curation.

## FUNDING INFORMATION

This research received no external funding.

## CONFLICT OF INTEREST STATEMENT

The authors have no competing interests.

## Supporting information


**Figure S1.** Outcome of the intention‐to‐treat population.
**Figure S2.** Outcome of short‐ and long‐term responders to pirtobrutinib.

**Table S1.** Selected clinical studies evaluating novel therapies in RS.

## Data Availability

Data are available upon request to the corresponding author.
