# Supplementary material for: Pirtobrutinib in Richter syndrome: Outcomes from the named patient programme in Italy
Source: Br J Haematol. 2026 Aug 30;209(5):2302–6. doi: 10.1111/bjh.70805 (PMC13748385; doi:10.1111/bjh.70805)
Supplement: Supplementary file 1 [file BJH-209-2302-s001.docx]

| **Therapeutic regimen** | **Study design** | **Patients**  **(N)** | **Median N**  **of prior therapies** | **Previous exposure to cBTKi, N (%)** | **≥1 prior line for RS, N (%)** | **ORR %** | **CR**  **%** | **Median PFS (months)** | **Median**  **OS (months)** |
| --- | --- | --- | --- | --- | --- | --- | --- | --- | --- |
| Pirtobrutinib  (our cohort) | Retrospective | 22 | 3.5 | 15/22 (68.2) | 19/22 (86.4) | 73 | 13 | 6.1 | 18.2 |
| Pirtobrutinib  (BRUIN phase 1/2)^8^ | Prospective | 82 | 4 | 61/82 (74.4) | 74/82 (90.2) | 50 | 13 | 3.7 | 12.5 |
| RICE + covalent BTKi^9^ | Retrospective | 11 | 1 CLL  0 RS | 5/11 (45.5) | 5/11 (45.5) | 91 | 64 | NR (12‑mo PFS 64%) | NR (12‑mo OS 73%) |
| Axicabtagene ciloleucel^10^ | Retrospective | 9 | 4 | 8/9 (88.9) | 5/9 (55.6) | 100 | 62 | NR | NR |
| Anti‑CD19 CAR‑T  (mixed products)^11^ | Retrospective | 69 | 4 | BTKi and/or BCL2i 58/69 (84.0) | NR | 63 | 46 | 4.7 | 8.5 |
| Nivolumab + ibrutinib^12^ | Prospective | 24 | 3 | 13/24 (54.2) | 10/24 (41.7) | 42 | 33 | NR | 13.0 |
| Atezolizumab + R‑GemOx^13^ | Prospective | 9 | 2 | NR | NR | 38 | 23 | 4.2 | 7.7 |
| Epcoritamab^14^ | Prospective | 42 | 2 CLL | 22/32 (69) CLL  6/21 (29) RS | 21 (50) | 48 | 40 | 3.0 | 13.0 |
| Mosunetuzumab^15^ | Prospective | 20 | 2.5 | 9/20 (45) | 20/20 (100) | 40 | 20 | 3.2 | 10.2 |

**Supplementary Table 1. Selected clinical studies evaluating novel therapies in RS**

Abbreviations: cBTKi: covalent BTK inhibitor; CLL: chronic lymphocytic leukemia; RS: Richter syndrome; ORR: overall response rate; CR: complete remission; PFS: progression free survival; DOR: duration of response; OS: overall survival; RICE: rituximab, ifosfamide, carboplatin, etoposide; GemOx: gemcitabine, oxaliplatin; NR: not reported.
