# Supplementary material for: Pirtobrutinib in Richter syndrome: Outcomes from the named patient programme in Italy
Source: Br J Haematol. 2026 Aug 30;209(5):2302–6. doi: 10.1111/bjh.70805 (PMC13748385; doi:10.1111/bjh.70805)
Supplement: Supplementary file 2 [file BJH-209-2302-s002.zip › bjh70805-sup-0002-DataS1.docx]

**Supplementary** **Figure S1. Outcome of the intention-to-treat population.** **A.** Progression-free survival (PFS). **B.** Overall survival (OS).

**Supplementary** **Figure S2. Outcome of short and long-term responders to pirtobrutinib. A.** Clinical course of a prototypical patient with short-lived response to pirtobrutinib (patient #18). Represented are peripheral blood mononuclear cell (PBMC) count, platelet (PLT) count and LDH values. Reference values for PBMC and PLT counts are on the left Y axis, those for LDH are on the right Y axis. **B.** Overall survival (OS) stratified for the duration of response to pirtobrutinib. **C.** OS comparing long-term responders versus short-lived and non-responders to pirtobrutinib.
